# Around the Hospitals

**Published:** 1892-04-30

**Authors:** 


### Around the Hospitals.

The **Bridgnorth and South Shropshire Infirmary** report for the year 1891 proves two things. First of all that no time should be lost in raising funds to erect an entirely new building, and secondly, that a rule should be at once passed to prohibit the Matron and nurse from leaving the hospital to attend to out-patients in their own homes. We have often given reasons against mixing the duties of district and hospital nurses, and we note with satisfaction that the Surgical Committee report that, "owing to the seriousness of some in-patient cases, and the absence of the second nurse, the number of visits paid by the Matron and nurse to the out-patients have been considerably less during the current year." Mr. Craig, the house surgeon, and Miss Hadfield, the Matron, are evidently doing good work at Bridgnorth at the present time.

The annual meeting of the **City of Dublin Hospital** proved a very lively and important one this year. The Master of the Rolls now fills the recently vacated office of Chairman, and the Earl of Pembroke has made a conditional promise of £6,000 towards the intended increase of accom modation, which is much needed, this being the only point of complaint under the last official inspection of the hospitals of Dublin. At the meeting Mr. Marcus Moses, a generous benefactor of the institution, pointed out that far from the support afforded to the hospital being derived from the districts outside Dublin, which benefited by its services, that in the wealthy township of Pembroke he had taken one of the principal roads containing fifty houses, whose inhabitants owned to incomes above £1,000, and on enquiry had found that but £5 only had been received from that district. Mr. Moses' experience could, unfortunately, be repeated in many other parts, and would probably put the wealthy inhabitants of many towns to the blush.

**The Royal Free Hospital**, Gray's Inn Road, must always possess special interest for the truly benevolent giver. It is and always has been free to the deserving when overtaken by accident or illness. The sixty-fourth annual report for 1891 affords evidence of the excellence of the present management. It has been determined to obtain a Royal Charter of Incorporation to enable it to acquire and hold land and to obviate the necessity of trustees. Such incorporation would relieve the members of the committee from personal liability from contracts and enable them to take legal proceedings when necessary in the name of the hospital. The importance of the Mortmain and Charitable Uses Act, 1891, to all hospitals is duly noted, but we are sorry to observe the omis sion of all mention of The Hospitals Association, which obtained this important concession for the charities of this country. A Ladies' Visiting Committee has been appointed, which has tended to secure improvements in the domestic arrangements whereby the comfort of both patients and nurses has been increased. The expenditure, which amounted to £11,000 exceeded the total income from all sources by upwards of £3,000, which large deficit we commend to the favourable consideration of our readers. It may be well to notice that £500 was received from private nursing and nearly £118 from nurses and probationers' fees, or together £618, against an expenditure of £763 in salaries to the nursing staff. The annual subscriptions only amounted to £1,083, and we should hope that they will be at least trebled during the next two years. The qualifications for annual and life Governors are respectively a subscription of one guinea and a donation of £10 10s. in one year. One thousand eight hundred and sixty-six in-patients, 12,847 outpatients, and 9,876 casualties, or altogether 24,589 patients were under treatment during 1891.

